# The Development of Visual Areas Depends Differently on Visual Experience

**DOI:** 10.1371/journal.pone.0053784

**Published:** 2013-01-07

**Authors:** Wen Qin, Yong Liu, Tianzi Jiang, Chunshui Yu

**Affiliations:** 1 Department of Radiology, Tianjin Medical University General Hospital, Tianjin, China; 2 LIAMA Center for Computational Medicine, National Laboratory of Pattern Recognition Institute of Automation, Chinese Academy of Sciences, Beijing, China; 3 Department of Radiology, Xuanwu Hospital, Capital Medical University, Beijing, China; University of Leuven, Belgium

## Abstract

Visual experience plays an important role in the development of the visual cortex; however, recent functional imaging studies have shown that the functional organization is preserved in several higher-tier visual areas in congenitally blind subjects, indicating that maturation of visual areas depend unequally on visual experience. In this study, we aim to validate this hypothesis using a multimodality MRI approach. We found increased cortical thickness in the congenitally blind was present in the early visual areas and absent in the higher-tier ones, suggesting that the structural development of the visual cortex depends hierarchically on visual experience. In congenitally blind subjects, the decreased resting-state functional connectivity with the primary somatosensory cortex was more prominent in the early visual areas than in the higher-tier ones and were more pronounced in the ventral stream than in the dorsal one, suggesting that the development of functional organization of the visual cortex also depends differently on visual experience. Moreover, congenitally blind subjects showed normal or increased functional connectivity between ipsilateral higher-tier and early visual areas, suggesting an indirect corticocortical pathway through which somatosenroy information can reach the early visual areas. These findings support our hypothesis that the development of visual areas depends differently on visual experience.

## Introduction

Visual deprivation is an idea model to investigate how visual cortex is reorganized to ‘perceive’ the outside world. Congenitally blind (CB) subjects who have lost visual experiences since birth have been frequently reported to have altered structural and functional characteristics in the visual cortex, including increased cortical thickness [1]–[3], brain regional homogeneity [4], metabolism and blood flow [5]–[8], decreased regional volume [9]–[11], white matter integrity [12], [13], and altered resting-state functional connectivity (rsFC) [14]. Moreover, converging evidence suggests that both the early and higher-tier visual areas in CB subjects are activated during performing a variety of tactile or auditory tasks [15], [16]. These findings indicate that visual experience plays an important role in shaping the structural and functional organization of the visual cortex during the process of development.

However, the notion of the visual cortex as a unimodal system molded only by visual experience has recently been challenged. In sighted subjects, tactile- or auditory-related tasks can activate the dorsal and ventral visual pathway [17]–[22] and MT+ [23], [24]. Furthermore, increasing evidence has confirmed that CB subjects preserved functional organization in several higher-tier visual areas when performing non-visual sensory task, such as the dorsal stream specialized for spatial processing [25], [26] and action control [27], the ventral stream for reading [28] and object recognition [21], [29], [30], and the MT+ for motion processing [23], [31]–[34]. It is more interesting to note that both CB and SC subjects using visual-to-auditory (or visual-to–tactile) sensory substitute devices (SSD) also recruited the ventral and dorsal visual pathway by both spatial and object related tasks, such as object recognition, motion, orientation and location detection, navigation, and so on [35]–[41]. Although the extent and magnitude of the activation of the recruited areas differed between sighted control (SC) and CB individuals [21], [23], the co-activation of the higher-tier visual cortex by non-visual sensory inputs in SC and CB highly indicates that the development of at least parts of their functional organization does not require visual experience [42], [43].

Although the structural and functional reorganization of the visual cortex have been frequently studied in CB, little is known on the differential effects of visual deprivation on the development of the structural and resting-state functional organization of the early and higher visual cortices. Based on the earlier findings, we hypothesize that the development of visual areas depends differently on visual experience. The early visual areas are mainly modulated by visual experience through their dense connections with the visual input system, but they can also respond to non-visual sensory inputs via sparse connections with those modalities [44]. Although the higher-tier visual areas receive visual inputs from the early ones, they also have dense connections with non-visual sensory modalities [45] and can be molded by non-visual sensory stimuli. In order to verify our hypothesis, we introduce a multimodality MRI approach, including cortical thickness and resting-state functional connectivity (rsFC) analyses. We predict that the cortical thickness of the early visual areas will be altered in CB subjects, whereas that of the higher-tier areas will be less affected or even normal. We also predict that the resting-state functional connectivity (rsFC) of the early visual areas with primary somatosensory cortex (S1) cannot develop normally in CB subjects, but those of the higher-tier ones will develop normally.

If our hypothesis is correct, the preserved rsFC between the higher-tier visual areas and non-visual sensory areas may explain the activation of these higher-tier visual areas in CB subjects by non-visual tasks [25], [26], [46]. However, it cannot explain why the early visual areas are also activated in the CB when performing these tasks. One possible mechanism is that the early visual areas may receive non-visual sensory information via the higher-tier ones that have normally developed rsFC with the non-visual sensory areas. This possibility facilitates the hypothesis that the rsFC between the higher-tier and early visual areas will develop normally in CB subjects.

## Materials and Methods

### Subjects

A total of 95 right-handed subjects comprising 39 CB (24 males, mean age 24.4±4.8 years) and 56 SC subjects (40 males; mean age 25.1±4.7 years) participated in this experiment. Group comparisons did not reveal any significant differences in either age (t = −0.64, P = 0.52) (by two-sample t-test) or gender (chi-square = 1.02, P = 0.31) (by chi-square test). All CB subjects had lost their sight since birth, and 20 of them had weak light perception. None of the CB subjects had experience on pattern vision, such as contours, shapes or orientation, etc. (Table 1). The protocol was approved by the Medical Research Ethics Committee of Tianjin Medical University General Hospital, and written informed consent was obtained from all participants prior to the experiment.

**Table 1:** Demographic information of congenitally blind subjects.

Subjects	Gender	Age (years)	Light perception	Causes of blindness
CB001	M	28	Weak	Retinal dysplasia
CB002	F	28	Weak	Retinal pigmentosa
CB003	F	27	None	Optic nerve atrophy
CB004	M	23	Weak	Fundus oculi illness
CB005	M	24	None	Eyeball dysplasia
CB006	F	27	Weak	Retinal pigmentosa
CB007	F	20	None	Unknown
CB008	M	22	None	Retinal dystrophia
CB009	M	30	Weak	Congenital cataract
CB010	M	22	Weak	Congenital cataract
CB011	M	27	None	Optic nerve atrophy
CB012	F	20	None	Microphthalmus
CB013	M	23	Weak	Unknown
CB014	M	39	None	Microphthalmus
CB015	M	36	Weak	Hypoplasia of fundus oculi
CB016	M	29	Weak	Unknown
CB017	F	21	Weak	Hypoplasia of fundus oculi
CB018	M	31	Weak	Congenital microphthalmus
CB019*	F	27	None	Hypoplasia of fundus oculi
CB020	F	27	None	Congenital cataract
CB021	M	28	None	Hypoplasia of fundus oculi
CB022	F	23	None	Retinitis pigmentosa
CB023	M	21	None	Retinitis pigmentosa
CB024	M	21	None	Optic nerve atrophy
CB025	M	25	None	Optic nerve atrophy
CB026	M	19	Weak	Retinitis pigmentosa
CB027	M	25	Weak	Retinitis pigmentosa
CB028	F	24	Weak	Optic hypoplasia
CB029	M	22	None	Congenital glaucoma
CB030	M	29	None	Optic nerve hypoplasia
CB031	M	23	None	Congenital glaucoma
CB032	F	27	Weak	Optic nerve atrophy
CB033	F	16	Weak	Optic nerve atrophy
CB034	F	18	Weak	Retinitis pigmentosa
CB035	F	22	None	Congenital cataract
CB036	F	22	Weak	Congenital glaucoma
CB037*	M	21	Weak	Congenital glaucoma
CB038	M	19	None	Optic nerve hypoplasia
CB039	M	19	None	Retrolental fibroplasia

* These two CB subjects were excluded from rest-state functional connectivity analysis for excessive head motion. CB  =  congenitally blind.

### MRI data acquisition

MRI data were obtained using a 3.0-Tesla MR scanner (Trio Tim system; Siemens, Erlangen, Germany) with a 12-channel head coil. Structural images were acquired using 3D magnetization-prepared rapid-acquisition gradient echo (MP-RAGE) sequences with the following parameters: repetition time (TR)/echo time (TE)/inversion time (TI)  = 2000/2.6/900 ms, flip angle  = 9°, matrix  = 256×224, field of view (FOV)  = 256 mm ×224 mm, 176 continuous sagittal slices with a 1-mm thickness. The resting-state fMRI data were acquired with a gradient-echo echo-planar imaging (GRE-EPI) sequence. The acquisition parameters included: TR/TE  = 2000/30 ms, flip angle  = 90°, matrix  = 64×64, FOV  = 220 mm ×220 mm, 32 axial slices with a 3-mm slice thickness and a 1-mm gap. During the fMRI scans, all subjects were instructed to keep their eyes closed, to relax, and to move as little as possible.

### Cortical thickness computation

Cortical thickness was calculated using Freesurfer V.5.1.0 [47], [48] (http://surfer.nmr.mgh.harvard.edu/). All procedures were performed using the automated surface-based pipeline with default parameters of the Freesurfer package, which mainly included segmentation, surface reconstruction, and surface-based spatial registration. First, the 3D T1 structural images were registered with the Talairach atlas [49], and the intensity variation in the white matter was removed by intensity normalization. The skull was stripped using a deformable template model [50]. White matter was then segmented based on intensity and neighbor constraints. Thereafter, the gray/white surface was obtained by tessellation of the gray/white matter boundary and topology correction. The pial surface was generated by nudging the gray/white matter surface along the T1 intensity gradients to reach the gray matter/cerebrospinal fluid boundary. Both surfaces were represented by vertices. The distance between the gray/white matter surface and its corresponding pial surface was defined as the cortical thickness [51]. To compare cortical thickness between groups, the cortical surface of each subject was transformed into an average surface space (fsaverage template, provided in Freesurfer package) using a spherical registration method [52]. Finally, the cortical thickness maps were smoothed with a Gaussian kernel of 10 mm full width at half maximum (FWHM).

### Resting-state functional connectivity analysis

The resting-state fMRI data were preprocessed using Statistical Parametric Mapping (SPM8, http://www.fil.ion.ucl.ac.uk/spm). The preprocessing steps included: (1) the first 10 volumes of blood oxygen level-dependent (BOLD) time series were removed for signal instability caused by T1 relaxation; (2) slice timing was performed to correct the acquisition time delay between slices within each volume; (3) the motion parameters were estimated, and each volume was realigned to the first volume (as a result, subjects CB019 and CB037 were excluded from further analysis because their translational or rotational parameters exceeded 2 mm or 2 degrees); (4) the remaining data set was normalized into the Montreal Neurological Institute (MNI) EPI template and resampled into 2×2×2 mm^3^ voxels; (5) the linear trend and several sources of spurious variances, including the estimated motion parameters, and average BOLD signals in the ventricular and white matter regions were removed from the data through linear regression; (6) a band-pass frequency filter (0.01–0.08 Hz) was applied to reduce low-frequency drift and high-frequency noise; (7) the filtered BOLD images were spatially smoothed by convolution with an isotropic Gaussian kernel (FWHM  = 6 mm).

The S1 was defined using the probabilistic map of Harvard-Oxford Cortical Structural Atlas (implemented in FSL package), voxels with probability higher than 50% were included. The region of interest (ROI) of each visual area was extracted using the Human PALS-12 atlas [53]. Because the PASL-12 atlas is population-averaged and surface-based, the surface ROIs were registrated into volumetric ones in MNI space that contain the voxels between white/gray matter surface and pial surface. We first computed the correlation coefficients between the mean time series of the S1 and that of each voxel of the whole visual cortex in a voxel-wise manner. Furthermore, we also calculated the correlation coefficients between the mean time series of the S1 and that of each visual ROI, and between every pair of visual ROIs. Before statistical analysis, the correlation coefficients were transformed into z-values using the Fisher r-to-z transformation to improve the normality.

### Statistical analysis

Group comparison of cortical thickness was performed in a vertex-wise manner using a general linear model (GLM) with age, gender and whole brain mean thickness as the nuisance covariates and within a mask bilaterally including the visual cortex and S1. A FDR method with a threshold of *P*<0.01 was selected to correct for multiple comparisons in combination with a cluster size threshold of *P*<0.05 (Monte Carlo simulations). Furthermore, the mean cortical thickness of each visual area of each subject was extracted based on the Human PALS-12 atlas. After regressing out age, gender and whole brain mean thickness effects, the ROI-based group comparisons were carried out using two-sample t-test at *P*<0.05 (Bonferroni corrected).

Voxel-based rsFC analyses between the S1 and the whole visual cortex were performed using an FDR threshold of *P*<0.01. First, individual z-map for each group (CB or SC) were entered into a random effect one-sample t-test in a voxel-wise manner to determine the visual areas that showed significant correlations with the seed ROIs. Then, a two-sample t-test was carried out to investigate group differences in the rsFC. To validate the results of the voxel-based rsFC analyses, we further compared the group differences in the rsFC between S1 and each visual area using a two-sample t-test after regressing out age and gender influences (*P*<0.05, Bonferroni corrected). Finally, we investigated inter-group differences in the rsFC between every two visual areas using a two-sample t-test after regressing out age and gender effects (*P*<0.05, Bonferroni corrected).

## Results

### Vertex-based cortical thickness analyses

**Figure 1:**
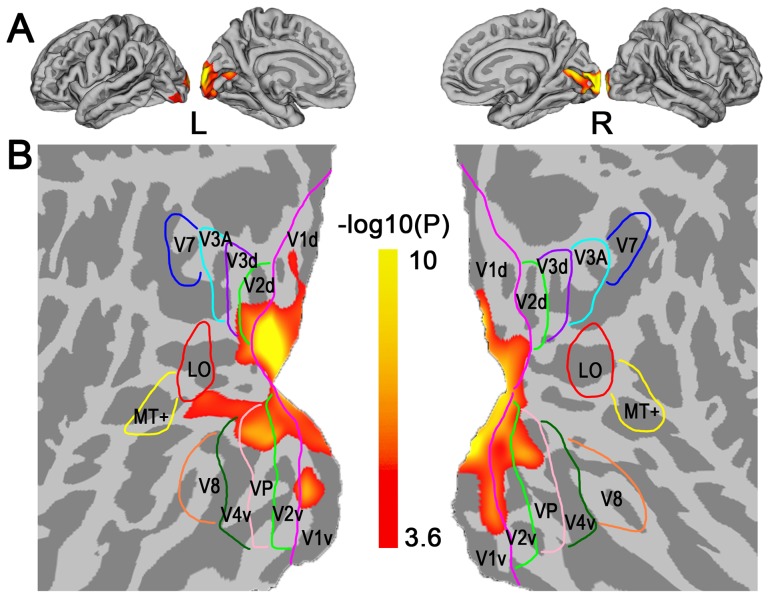
Comparison maps of cortical thickness between CB and SC subjects in a vertex-wise manner. ***A***, significantly increased cortical thickness in CB subjects is observed in the visual areas while absent in primary somatosensory cortex (S1) within a searching mask including the whole visual cortex and S1 (*P*<0.01, FDR corrected). ***B***, significantly increased cortical thickness in CB subjects is observed in the early visual areas (V1, V2 and VP), but not in higher-tier ones (V3A, V7, MT+ and V8) and in S1. Scale bar represents the log-transformed *P* value.

Compared with SC subjects, CB individuals showed significantly increased cortical thickness in the early visual areas (*P*<0.01, FDR corrected); however, most of the higher-tier visual areas, especially the dorsal visual stream, did not show any significant changes in cortical thickness (Fig. 1). No significant group differences were found in cortical thickness in S1 even using a loose threshold (*P*<0.05, uncorrected). To investigate whether weak light perception affected the cortical thickness analysis results, we also compared the cortical thickness between CB subjects with and without light perception. There was no significant group difference even using an uncorrected *P*<0.05.

### ROI-based cortical thickness analyses

**Figure 2:**
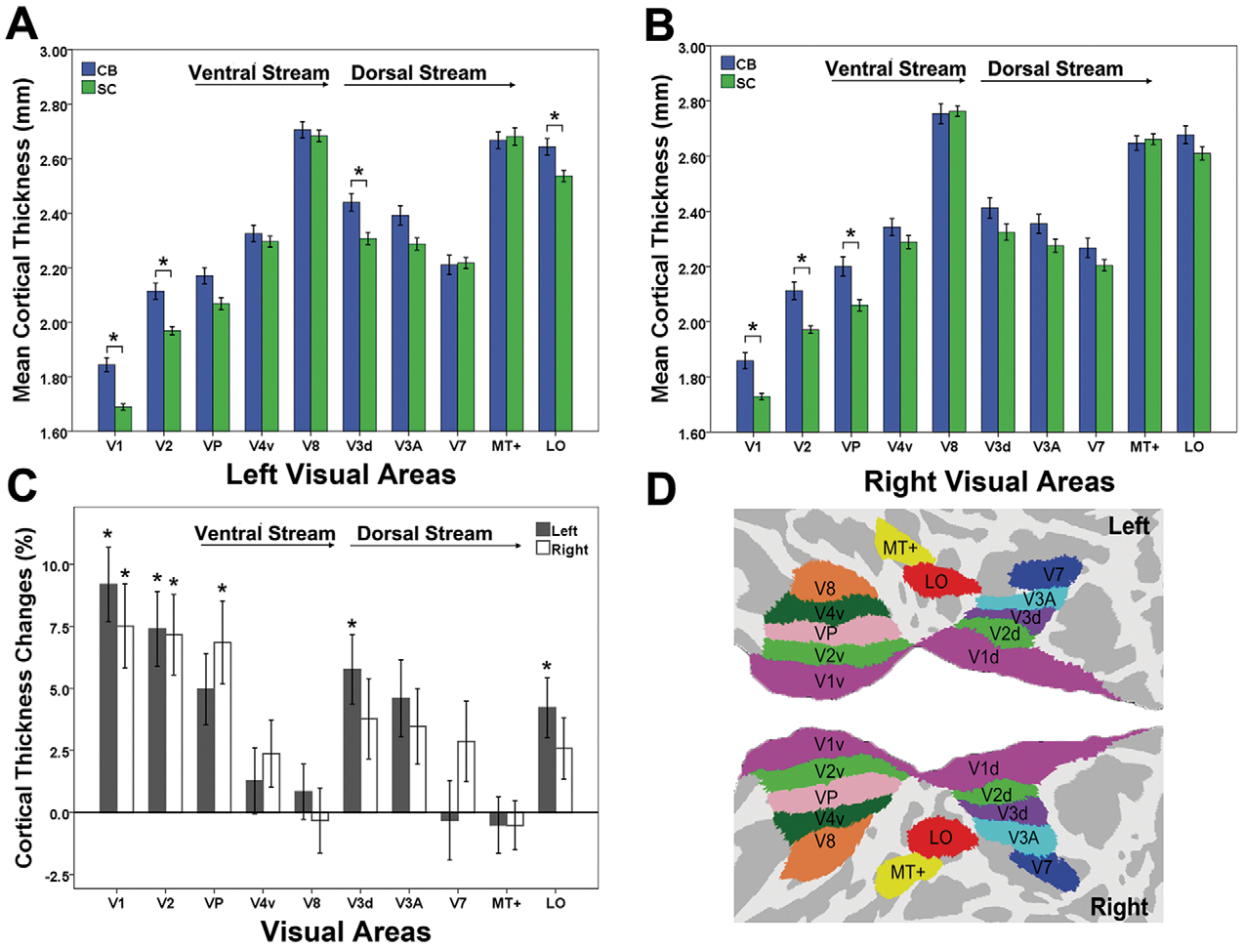
ROI-based analyses of the cortical thickness between CB and SC subjects. An asterisk (*) represents significant difference between groups. ***A*** and ***B***, significant increases in cortical thickness are found in the left V1, V2, V3d and LO, and in the right V1, V2 and VP (*P*<0.05, Bonferroni corrected). ***C***, percent changes of cortical thickness in CB subjects relative to SC subjects. The cortical thickness is significantly increased in the early visual areas (V1, V2, VP and V3d), but not in several higher-tier visual areas (V3A, V7, MT+ and V8) in CB subjects. ***D***, visual areas derived from the Human PALS-12 atlas.

To further elucidate the specific change in cortical thickness of each visual area, we extracted the mean cortical thickness of each visual area in each subject based on the Human PALS-12 atlas (Fig. 2*D*). In the left hemisphere, CB subjects showed significantly increased cortical thickness relative to SC subjects in V1, V2, V3d and LO (*P*<0.05, Bonferroni corrected) (Fig. 2*A*). Similarly, in the right hemisphere, CB subjects had significantly increased cortical thickness in V1, V2 and VP (*P*<0.05, Bonferroni corrected) (Fig. 2*B*). The mean increase amplitude in cortical thickness of CB subjects versus SC subjects was 8% in V1, 7% in V2, 6% in VP, 5% in V3d, 4% in V3A, and 3% in the LO (Fig. 2*C*). The remaining higher-tier visual areas did not show any significant group differences in cortical thickness even using an uncorrected *P*<0.05.

### Voxel-based rsFC analyses between S1 and visual areas

**Figure 3:**
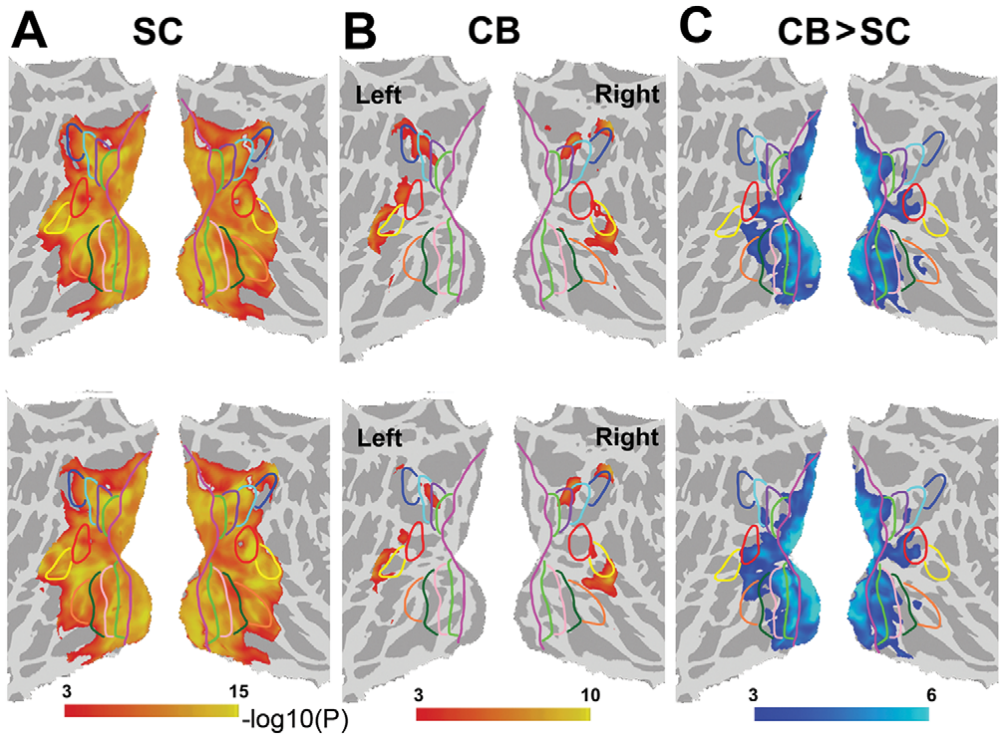
The rsFC maps between the S1 and the visual areas. ***A***, the rsFC map of SC subjects. ***B***, the rsFC map of CB subjects. ***C***, the differential maps of rsFC between CB and SC subjects with thresholded at P<0.05 (FDR corrected). The upper and lower panel represents the FC results of the S1 area in the left and right hemisphere, respectively. Compared with SC subjects, CB subjects showed significantly decreased (cool color) rsFC between the S1 and the visual areas, especially the early visual areas (V1 and V2) and the ventral stream (VP and V4v).

The S1 of SC subjects showed positive rsFC with most of the visual areas (Fig. 3*A*) (*P*<0.01, FDR corrected). In contrast, the S1 of CB subjects had positive rsFC with only parts of the higher-tier visual areas (*P*<0.01, FDR corrected) (Fig. 3*B*). Group comparison showed that CB subjects had significantly decreased rsFC between non-visual sensory ROIs and visual areas relative to SC subjects (Fig. 3*C*). Moreover, the early visual areas were more pronouncedly affected than the higher-tier ones, and the ventral stream was more pronouncedly affected than the dorsal one.

### ROI-based rsFC analyses between S1 and visual areas

**Figure 4:**
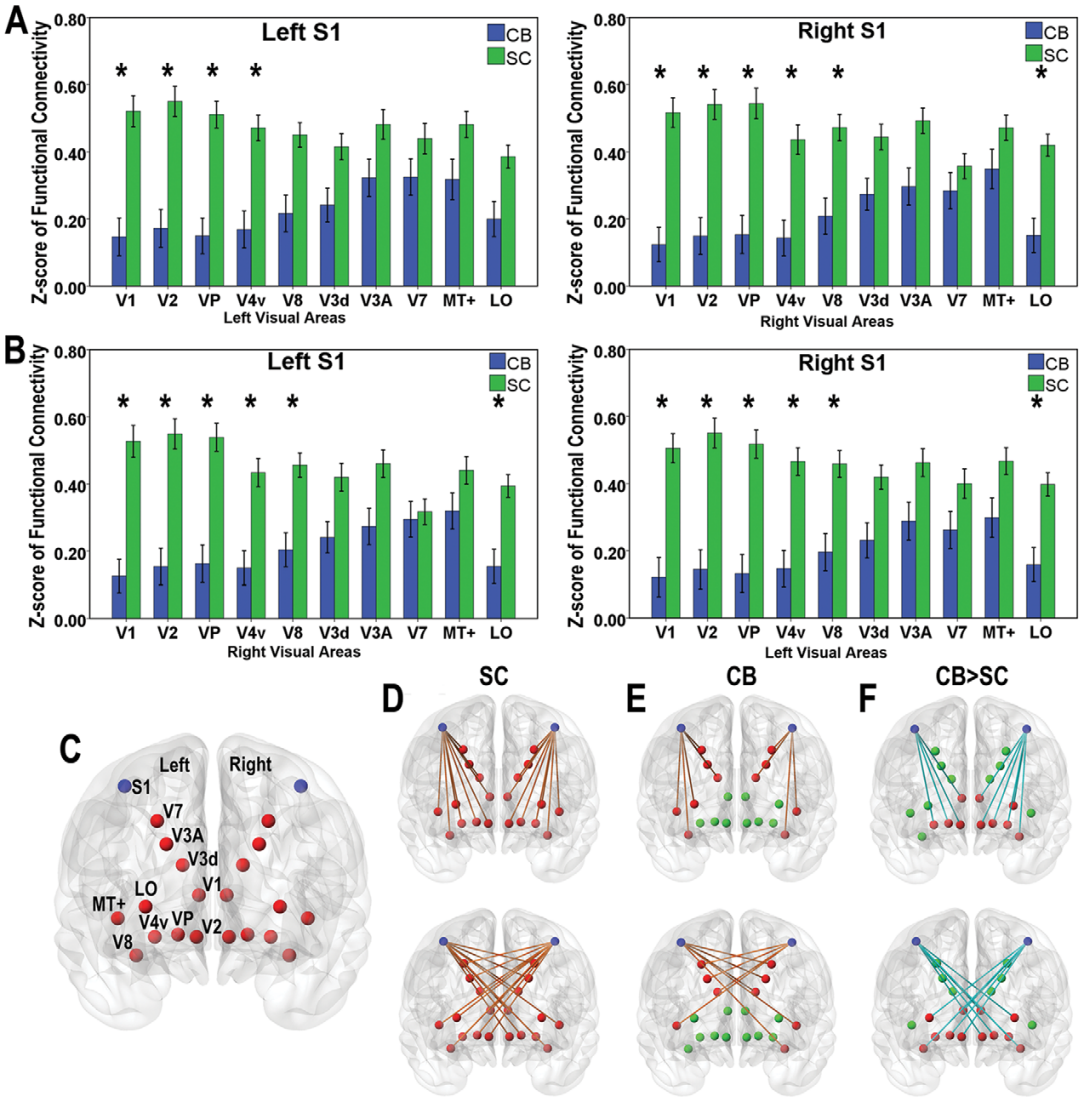
ROI analyses of the rsFC between the S1 and visual areas. ***A*** and ***B***, the mean rsFC of CB (blue bar) and SC (green bar) subjects between intra-hemisphere and intra-hemisphere, respectively. An asterisk represents significantly differences (*P*<0.05, Bonferroni corrected). ***C***, the defined S1 and visual ROIs. ***D***, rsFC patterns between S1 and visual areas in the SC. ***E***, rsFC patterns between S1 and visual areas in the CB. ***F***, group differences of rsFC between CB and SC subjects. Orange and light blue edges represent positive and negative effects. Blue, red and green nodes represent S1, affected and non-affected visual areas. Compared with SC subjects, CB subjects showed significantly decreased rsFC of the S1 with the early visual areas and the ventral stream ones, with no difference being observed in several higher-tier visual areas.

We further computed the rsFC between S1 and each visual ROI. Compared with those of SC subjects, the S1 of the CB subjects showed significantly decreased rsFC with the early visual areas (V1 and V2) and the ventral stream (VP, V4v and V8), but not with several higher-tier visual areas in the dorsal visual stream (V3d, V3A, V7 and MT+), using a threshold of *P*<0.05 (Bonferroni corrected) (Fig. 4). The hierarchical trend that the early visual areas are more affected than the higher-tier ones is also present in rsFC pattern in CB.

### ROI-based rsFC analysis within the visual areas

**Figure 5:**
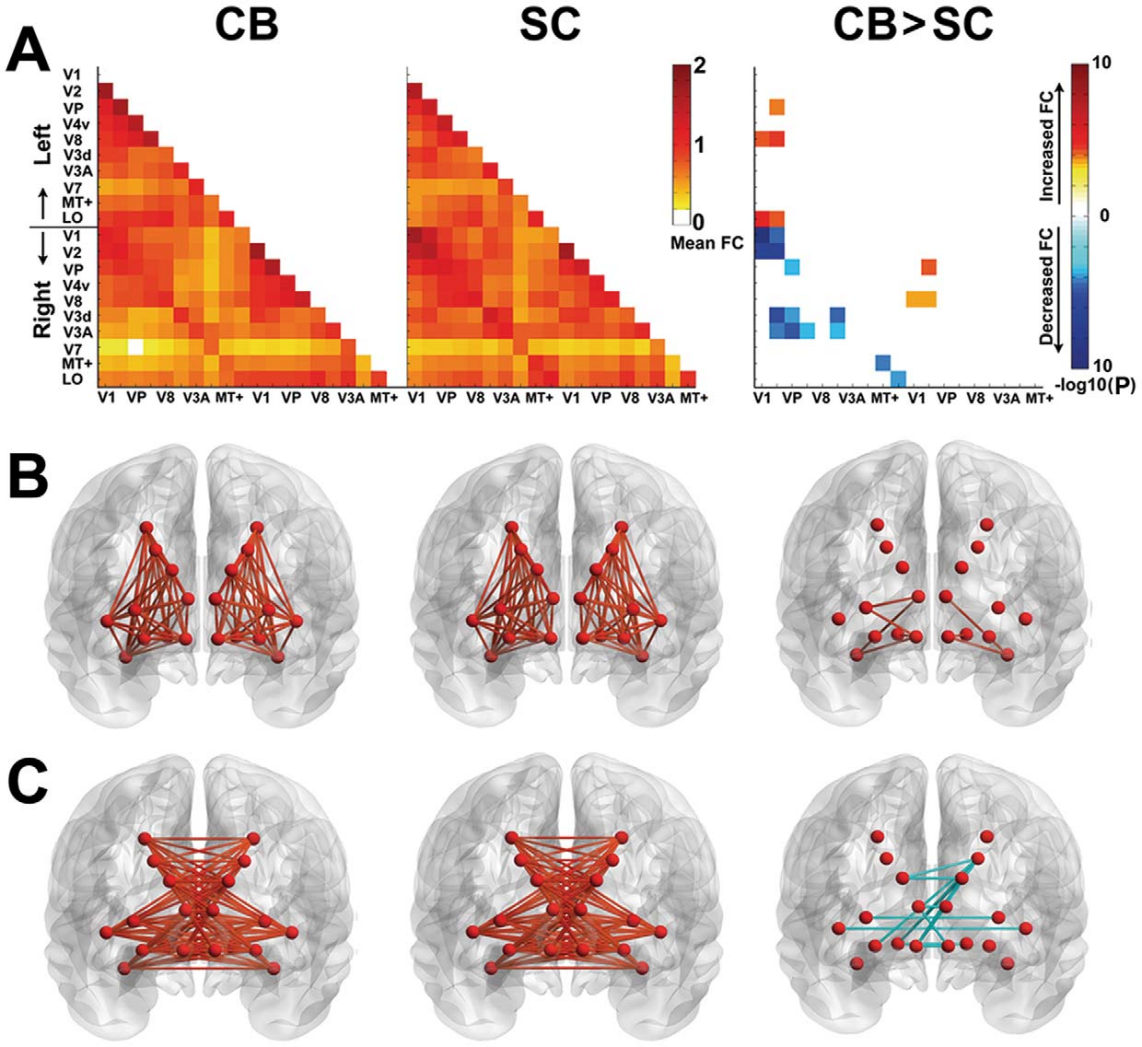
The rsFC patterns within the visual areas in CB and SC subjects. ***A***, the mean rsFC in CB and SC subjects, and group comparisons (*P*<0.05, Bonferroni corrected), in which hot and cool colors represent positive and negative effects, respectively. Color bar represents the log-transformed *P* value. ***B***, the rsFC patterns between each pair of the ipsilateral visual areas. ***C***, The rsFC patterns between each pair of inter-hemispheric visual areas. Orange and light blue lines represent increased and decreased rsFC. Both CB and SC subjects show positive rsFC between visual areas. Significantly increased rsFC between the early (V1 and V2) and several ipsilateral higher-tier visual areas (V8 and LO), while decreased rsFC between inter-hemispheric visual areas are shown in CB subjects.

We investigated the rsFC between each pair of the visual areas and found significantly increased rsFC (*P*<0.05, Bonferroni corrected) between the early visual areas (V1 and V2) and several higher-tier visual areas (V8 and LO) in the same hemisphere in CB subjects (Fig. 5). We also found decreased rsFC between several inter-hemispheric visual areas (Fig. 5).

## Discussion

In this study, combined structural and functional analyses, we found CB subjects showed significantly increased cortical thickness in the early visual areas while absent in the higher-tier ones; furthermore, the decreased rsFC with the S1 was more prominent in the early visual areas than in the higher-tier ones and were more pronounced in the ventral visual stream than in the dorsal one, suggesting that the development of the structural and functional organization of the visual cortex depends differently on visual experience. Moreover, CB subjects showed normal or increased rsFC between the higher-tier and early visual areas in the ipsilateral hemisphere, suggesting an indirect corticocortical pathway through which non-visual sensory information can reach the early visual areas.

### The structural and functional reorganization in CB

It is important to note that evidence of reorganization in the visual cortex in CB comes from studies with different modalities. How to reconcile findings from these modalities is critical for understanding the neural mechanisms of the altered cortical organization in CB. For example, the contradictory findings of the increased cortical thickness [1], [2] and decreased gray matter volume (GMV) [9] in the early visual cortex in the early blind could be explained by the overly reduced surface area (Jiang et al., 2009). Similarly, the unchanged cortical thickness [1] and decreased GMV [54] in the visual cortex in the late-onset blind could also be driven by reduced surface area (Jiang et al., 2009).

The following factors may affect the morphology of the early visual cortex in CB. Firstly, the synaptic pruning of the visual cortex is dependent on visual experience [55], [56] and is interfered in the CB. Thus the relatively high synaptic density may contribute to the thickened early visual cortex in CB [1], [2]. However, these “redundant” synapses cannot explain the recruitment of early visual cortex in a variety of non-visual tasks in CB [15], [16]. Secondly, early visual deprivation really results in the increases in corticocortical [57], [58], thalamocortical [57], [59] and intracortical connections [60], [61] in the visual areas. These increased connections may contribute to the increased cortical thickness in the CB and may form the structural basis for the visual cortex responsing to non-visual stimuli. Finally, axonal degeneration secondary to the peripheral damage of the anterior visual pathway will result in the atrophy of the gray and white ma motion radiation [9], [13] in CB subjects. The atrophied white matter of the visual area may account for the reduction of the surface area in the CB. Therefore, the neural mechanisms of the thickened visual cortex in the CB are complex and represent a final consequence of multiple factors because increased synaptic density contributes to the increased cortical thickness whereas axonal degeneration lead to the decrease in cortical thickness.

Different functional connectivity (FC) patterns between the early visual cortex and non-visual sensory cortex have been reported during resting-state and task-state in the CB. The early blind subjects exhibited increased FC between the S1 and early visual cortex during the stimulation of the S1 [62] and demonstrated increased effective connectivity from the primary auditory cortex to V1 when performing an auditory discrimination task [63]. During resting-state, however, decreased rsFCs between the V1 and non-visual sensory cortex have been frequently reported [14], [64], [65]. Although there is a close relationship in the FC patterns between resting-state and task-state [66], it does not mean that they share the same FC patterns. For example, significant differences in FC intensity between the fusiform gyrus and calcarine sulcus have been found under different task conditions [67]. The effective connectivity that measures the causal flow of one node to another is substantially different with functional connectivity that represents the simultaneous coupling of two nodes [68]. These factors might explain the different connectivity changes in CB under rest-state and task-state. The decreased rsFC only reflects the decoupling of brain activity between the early visual cortex and S1; however, it cannot simply imply the poor performance in handling tactile information, neither the poor activation in the occipital cortex.

The surprising findings with dramatically decreased resting-state FC between LO and S1 is difficult to explain the increased recruitments of this area in tactile processing in the CB, which had been confirmed by several previous task-evoked studies. In fact, not only the LO, but also the ventral pathway areas such as V4/V8 also encountered the same dilemma. Besides the difference in functional representation between task and rest-state as stated above, another possibility is that the tactile signals might reach the LO C and ventral visual areas via other indirect pathways. For example, increased resting-state FCs between occipital cortex and the prefrontal cortex have been reported by several research groups [14], [69]. As a result, the strengthened frontal-occipital FC might convey attention- (or working memory) modulated non-visual sensory signals to these object-related occipital areas for further manipulation. However, this hypothesis needs to be validated by further studies.

It has been suggested that the intrinsic functional connectivity reflects monosynaptic or polysynaptic connections [70], [71]. Bilateral visual cortices were anatomically connected by fibers of the splenium of corpus callosum [72]. Consequently, our finding of the decreased rsFC between inter-hemispheric visual areas might be as a result of the disrupted white matter integrity of these fibers [3], [12].

### The development of the visual cortex depends differently on visual experience

As mentioned in the introduction, the activation of the visual cortex by non-visual sensory stimuli both in SC and CB indicates that the visual cortex is supramodal in nature, and the development of the functional architecture in the higher visual cortex does not require visual experience to some extent (for reviews, see [42], [43]). In the present study, we found relative normally developed cortical thickness and rsFC in the higher visual cortex, especially in the dorsal pathway, which provided another aspect of evidence to support this concept. Furthermore, the present study provides converging evidence suggesting that the development of the visual areas depends differently on visual experience. Specifically, the early visual areas depend more on visual experience than the higher-tier ones, and the ventral stream visual areas depend more on visual experience than the dorsal ones. The early visual areas of sighted persons and animals receive sensory inputs primarily from the visual system and may also receive some direct or indirect inputs from non-visual sensory modalities (for reviews, see [44], [45], [57], [73]). However, the direct connections between early sensory areas of different modalities seem relatively sparse, and their functions remain unclear [44]. In contrast, the higher-tier visual areas have dense connections with non-visual sensory areas and have many feedback connections with the early visual areas [45]. The specific connection patterns of the early and higher-tier visual areas may determine their dependence on sensory experiences. The early visual areas are heavily connected with the visual thalamus but sparsely connected with non-visual sensory areas. As a result, these areas depend heavily on visual experience and less on non-visual inputs. The higher-tier visual areas have more connections with non-visual sensory areas than the early ones, and their development can be shaped by these non-visual sensory experiences. However, future studies are required to determine whether this mechanism explains the differential effects of visual experience on the ventral and dorsal visual streams.

It should be noted that differences in maturation rate and their susceptibility to visual deprivation between different visual areas might be another important factor that affects the structural and functional reorganization after early visual deprivation. For example, the sensitive period for normal maturation and susceptibility to damage of global motion (mediated primarily by the higher visual areas such as the MT+) appears to be much earlier and shorter than the sensitive period for those of visual acuity (mediated primarily by the early visual areas) [74]. The development of the motion and action ability (mediated by the dorsal visual stream) was found to be much earlier than that of the contour and form perceptual ability (mediated by the ventral visual stream) [75]–[78]. Earlier development of dorsal than ventral stream had also been validated by physiological and anatomical findings [79]–[82]. The relatively late developed visual areas (early visual areas and ventral stream) are more susceptive to early visual deprivation, which can explain our findings of the prominent structural and functional alterations in these visual areas.

### The neural pathway from non-visual sensory areas to the visual cortex

Because both the early and higher-tier visual areas can respond to tactile and auditory stimuli in CB subjects, it is important to understand how non-visual sensory information reaches the visual areas, especially the early ones. A tentative hypothesis is that the visual areas receive non-visual information through rewired thalamocortical connections [57], [59], [83], i.e., the lateral geniculate nucleus receives rewired non-visual projections from the auditory and somatosensory thalamic nuclei and then projects fibers to the visual areas in CB subjects. However, this hypothesis is not supported by findings of atrophy in the lateral geniculate nucleus and in its output projections [10], [12], [13]. An alternative hypothesis is that the visual areas receive non-visual sensory information through corticocortical connections between these sensory modalities [45], [84], which is supported by task-based fMRI studies [85], [86], and a resting-state fMRI study [87] in sighted subjects, an effective connectivity study [63], and transcranial magnetic stimulation (TMS) studies in blind humans [58], [62], [88], [89]. Furthermore, these corticocortical connections can be subdivided into direct long-range connections and indirect connections via intervening multimodal association areas. The former has been found in immature cats and hamsters and mature primates [45], [57], [90], while the latter is found in primates [91] and is confirmed by an effective connectivity study in humans [92]. Our findings of decreased rsFC between the early visual areas and S1 and the normal or even increased rsFC between the higher-tier visual areas and S1 and between the early and higher-tier visual areas in the same hemisphere in CB subjects would support the hypothesis wherein indirect corticocortical connections transmit non-visual sensory information to the early visual areas. Specifically, in the CB, non-visual sensory areas could receive tactile or auditory information and then transmit it to the early visual areas via higher-tier ones. It should be noted that this hypothesis needs further confirmation, although it seems to be supported by two effective connectivity studies [63], [92].

### Issues regarding to functional preservation and plasticity in occipital cortex

In sighted individuals, visual information is hierarchically processed from V1 to higher-tier visual areas along the ventral and dorsal pathways; however, this visual flow is interrupted by early visual deprivation. In contrast to the relative preserved functional preferences in the extrastriate areas after visual deprivation, V1 seems to be specially evoked by higher-order cognitive tasks such as memory [93], [94] and language [65], [95]–[97]. The preserved functional preferences are supported by our findings of the relatively unchanged functional connectivity between the extrastriate areas and S1. Furthermore, the decreased FC between early visual areas and S1 does not support direct tactile signal processing in V1; instead, this finding indicates that V1 might process higher cognitive-demanding functions, as revealed by previous task-driven results. Finally, as indicated by previous findings and present study, the intact indirect corticocortical pathways through which peripheral signals transmit from non-visual sensory areas to the “higher-tier” extrastriate areas and then to V1 is consistent with hypothesis by Buchel [98] that the cortical hierarchy in the occipital cortex might be reversed after early visual deprivation, such that extrastriate areas feed into V1, while V1 becomes a higher-tier area capable of processing multiple cognitive functions.

In summary, based on the cortical thickness and rsFC analyses in CB subjects, we propose that the development of visual areas depends differently on visual experience, which can explain most previous structural and functional findings in the visual areas of both sighted and blind subjects. Based on the rsFC analyses, we also suggest that the indirect corticocortical connection is a possible pathway through which non-visual sensory information could reach the early visual areas.
